# Concurrence of High Fat Diet and APOE Gene Induces Allele Specific Metabolic and Mental Stress Changes in a Mouse Model of Alzheimer’s Disease

**DOI:** 10.3389/fnbeh.2016.00170

**Published:** 2016-09-05

**Authors:** Yifat Segev, Adva Livne, Meshi Mints, Kobi Rosenblum

**Affiliations:** ^1^Sagol Department of Neurobiology, University of HaifaHaifa, Israel; ^2^Center for Gene Manipulation in the Brain, University of HaifaHaifa, Israel

**Keywords:** ApoE4, metabolic stress, high-fat diet, Alzheimer’s disease (AD), ATF4, translation regulation

## Abstract

Aging is the main risk factor for neurodegenerative diseases, including Alzheimer’s disease (AD). However, evidence indicates that the pathological process begins long before actual cognitive or pathological symptoms are apparent. The long asymptomatic phase and complex integration between genetic, environmental and metabolic factors make it one of the most challenging diseases to understand and cure. In the present study, we asked whether an environmental factor such as high-fat (HF) diet would synergize with a genetic factor to affect the metabolic and cognitive state in the Apolipoprotein E (ApoE4) mouse model of AD. Our data suggest that a HF diet induces diabetes mellitus (DM)-like metabolism in ApoE4 mice, as well as changes in β-site amyloid precursor protein-cleaving enzyme 1 (BACE1) protein levels between the two ApoE strains. Furthermore, HF diet induces anxiety in this AD mouse model. Our results suggest that young ApoE4 carriers are prone to psychological stress and metabolic abnormalities related to AD, which can easily be triggered via HF nutrition.

## Introduction

Aging is the main risk factor for Alzheimer’s disease (AD), which is a complex multifactorial disease involving both genetic and environmental factors. AD affects about 40 million people worldwide, with predictions for a dramatic increase in the coming years with the increase in the aged population (Dacks et al., 2014).

The most common form of the disease, sporadic, late onset AD, strikes on average after the age of 65, and is strongly linked to the Apolipoprotein E (ApoE) gene on chromosome 19. ApoE encodes a 299-amino acid, 34-kDa glycoprotein most prevalent in the liver, followed by the brain. ApoE mediates the transport and clearance of cholesterol, triglycerides, and other lipids (Li et al., 1988). The three human ApoE isoforms ɛ2, ɛ3 and ɛ4 differ from one another at residues 112 and 158, affecting their 3-dimensional structure. The ɛ4 allele has approximately 20% frequency in the general pooled population and roughly 50% in patients with AD, however, there is substantial heterogeneity in these prevalence estimates (Ward et al., 2012). The above finding renders ApoE4 the major genetic risk factor for sporadic AD (Rall et al., 1982; Strittmatter and Roses, 1996; Ward et al., 2012).

Different pathological mechanisms have been proposed to explain the effect of ApoE4, including mainly defects in the clearance of the beta-amyloid (Aβ) peptide, lipid and cholesterol metabolism, inflammation, mitochondrial dysfunction, neuronal function, and to a lesser extent, amyloid precursor protein (APP) cleavage (Mahley and Rall, 2000; He et al., 2007; Castellano et al., 2011; Chen et al., 2012; Dafnis et al., 2012). In addition, diabetes mellitus (DM) was suggested to exacerbate the risk of AD associated with ApoE4, as patients with diabetes who carry the ApoE4 allele are twofold more likely to develop AD than non-diabetic ApoE4 carriers (Peila et al., 2002). A possible mechanism specifically linking insulin resistance to AD in ApoE4 carriers is that Aβ is in part cleared by the insulin degrading enzyme (IDE). AD patients carrying the ApoE4 allele have been reported to express reduced IDE protein and mRNA levels in the hippocampus (Biessels et al., 2005; Schipper, 2011), suggesting a causal link between impaired insulin metabolism, either hypoinsulinemia or insulin resistance, and the pathogenesis of AD (Schipper, 2011).

Diverse metabolic stresses such as described above are mirrored in part by changes in the phosphorylation of eukaryotic initiation factor 2 (eIF2α) at serine 51 in cells and specifically in neurons, leading to changes in translation regulation (Sonenberg et al., 2000). At the same time, inverse correlation has been identified between eIF2α phosphorylation and memory or synaptic plasticity consolidation (Costa-Mattioli et al., 2007). Several lines of evidence indicate that phosphorylation of eIF2α or activation of its kinase, double-stranded RNA-activated protein (PKR), are involved in the pathogenesis of AD (Chang et al., 2002; Page et al., 2006). We have previously shown that ApoE4 AD model mice express high phosphorylation levels of eIF2α in the hippocampus compared to their age matched ApoE3 controls as early as 4 months of age (Segev et al., 2013). Furthermore, this increased phosphorylation is correlated with increased mRNA expression of activating transcription factor4 (ATF4) mRNA both in these mice and in postmortem AD brains (Segev et al., 2015).

Genome-wide association studies identified ApoE4 as the only significant gene associated with age-related cognitive decline in humans (De Jager et al., 2012). Additional studies suggest that ApoE4 has a detrimental effect on cognition before the typical signs of AD are apparent (Caselli et al., 2009). Supporting the above is the fact that we and others have shown that glial fibrillary acidic protein (GFAP)-ApoE4 expressing human-ApoE4 in astrocytes under the control of a GFAP promoter, as well as transgenic hAPP-ApoE4 and human-ApoE4 mice carrying the human ApoE4 gene under the endogenous ApoE mouse promoter, are impaired in spatial memory compared with their ApoE3 controls (Raber et al., 2000; Hartman et al., 2001; Kornecook et al., 2010; Salomon-Zimri et al., 2014; Segev et al., 2015). However, another study (Olsen et al., 2012) showed no memory deterioration in human-ApoE4 mice in the contextual memory of Fear Conditioning. These conflicting results may be due to the different mouse strains, but more likely due to the different protocols used. While Olsen and colleagues used a relatively strong protocol of two shocks, we used a single shock paradigm (Olsen et al., 2012; Segev et al., 2013). In our experience, a stronger shock makes it hard to detect cognitive differences between the mouse strains at a young age, therefore to detect changes in memory one needs to use a weaker protocol.

An additional factor linking metabolic stress to AD, is the β-site APP-cleaving enzyme 1 (BACE1). BACE1 is the initiator enzyme for the formation of Aβ, and surprisingly, increased phosphorylation of eIF2α as a result of metabolic stress, while decreasing general protein synthesis, increases a subset of mRNAs such as ATF4 (Costa-Mattioli et al., 2007) and BACE1 (O’Connor et al., 2008). Indeed, increased BACE1 protein levels and activity have been reported in the brains from AD patients (Fukumoto et al., 2002). In addition, BACE1 has been studied in relation to insulin deficiency and obesity. For example, a study by Devi et al. (2012) suggested that translational mechanisms through phosphorylation of eIF2α may underlie the upregulation of BACE1 associated with insulin deficient diabetes. Another group showed that mice lacking BACE1 are lean, resistant to diet-induced obesity and display increased peripheral tissue insulin sensitivity with improved whole-body glucose disposal (Meakin et al., 2012).

In addition to the known symptoms of cognitive impairment, increased anxiety may occur in up to 70% of AD patients during the course of their illness (Ferretti et al., 2001), and specifically patients with ε4/ε4 show higher anxiety scores than those with ε3/ε3 (Robertson et al., 2005).

In the present study, we tested the hypothesis that a high-fat (HF) diet converges on the ApoE4 allele, resulting in enhanced attenuation of cognitive abilities and metabolic phenotype. In order to test this hypothesis, we provided human-ApoE4 transgenic mice, which express the human ApoE gene physiologically under the murine ApoE promoter, and their ApoE3 controls with a HF diet for 20 weeks post- weaning, after which they were evaluated for metabolic and behavioral phenotypes. In agreement with others, we show that although ApoE4 mice gain less weight over time (Huebbe et al., 2015), they develop DM-like features (Arbones-Mainar et al., 2008; Pendse et al., 2009). Interestingly, consumption of the HF diet induces higher anxiety levels in the HF-apoE4 mice compared with HF-ApoE3 controls. Furthermore, BACE1 protein levels are increased in the hippocampus of HF-ApoE4 mice. Our findings point out the importance of convergence of environmental and genetic factors in the etiology and progression of AD at a young age, before pathological symptoms are apparent, and propose for further pursuing this hypothesis for additional and broader studies.

## Materials and Methods

### Animals

Humanized knock-in ApoE3 (B6.129P2-ApoE^tm2(ApoE*3)Mae^ N8) and knock-in ApoE4 (B6.129P2-ApoE^tm3(ApoE*4)Mae^ N8) homozygous male mice were supplied by Taconic (Hudson, NY, USA). All cages were placed in a light and temperature controlled room, and behavioral tests were conducted during daylight hours. All animals were handled in accordance with the University of Haifa regulations and the National Institutes of Health Guidelines (NIH publication number 8023), and maintained in a pathogen-free environment.

### Animal Treatment

All animal care and procedures were carried out in accordance with local guidelines and approved by the Animal Care and Use Committee of the University of Haifa. All efforts were made to minimize animal suffering and discomfort.

One month old male mice, both ApoE3 and ApoE4, were weaned, group housed in cages consisting of two or three littermates and given immediately a diet consisting of 60% fat (“high fat diet”, TD.06414, Harlan, WI, USA) *ad libitum* for a total of 3 months. Animals were weighed using lab scales and food intake was inspected on a weekly basis as the difference in mass between what was given at feeding time and the amount that was left 1 week later. The same animals were used for all experiments to assess the influence of the HF diet on the behavior of the different strains. Animals were sacrificed by cervical dislocation 2 weeks after the restraint test.

### Metabolic Measurements and Plasma Collection

Peripheral blood glucose was measured using an Accu-chek Performa glucometer (Roche, Switzerland) after nicking the tail tip. Non-fasting glucose levels were measured between 10 and 11:30 AM. Mice were deprived of food overnight for 12 h for fasting insulin measurements. Peripheral blood for insulin was collected by cardiac puncturing, after which mice were sacrificed. Blood was then diluted in 0.5 M EDTA and centrifuged for 25 min at 4°C and 3500 RPM. Plasma Insulin was determined using Rat/mouse Insulin Elisa kit #EZRMI-13K (Merck Millipore, Germany) according to the manufacturer’s instructions.

### Fear Conditioning

Fear conditioning was conducted in model chambers, measuring (25 × 25 × 25) cm internally (Panlab Harvard Apparatus, Barcelona, Spain). Each chamber was located inside a larger, insulated plastic cabinet that excluded external light and noise. The system enabled recording and analysis of the signal generated by animal movement via a high-sensitivity weight transducer system. Mice were placed in a chamber (context A with light [20 W bulb] and a 16-bar metal grid floor) for 120 s, after which the mice received a 2.9 kHz tone, applied for 30 s at 80 dB (conditioned stimuli; CS) and a subsequent 0.6 mA shock applied for 2 s (unconditioned stimuli; US), after which they remained in the chamber for 60 additional seconds and were returned to their home cage. The chambers were cleaned with 10% ethanol between successive sets of mice. Animal behavior was recorded, and the data were analyzed by Freeze Frame 3.0 software (Coulbourn Instruments, Whitehall, PA, USA). The indication for fear memory was percentage of time spent freezing.

### Elevated Plus Maze

The elevated plus maze (EPM) paradigm was conducted in a standard plus maze. The maze apparatus consisted of four arms in a cross position from a neutral central square. The horizontal arms are defined as “closed arms” (52 cm × 7 cm × 15 cm) and the vertical arms are defined as “open arms” and have no protective walls (52 cm × 7 cm). The 50 cm high maze was placed on a table in a room with indirect light. At the beginning of a 10 min test session, each mouse was placed in the middle of the maze facing the open arms and movement was monitored and registered by a camera system connected to an Ethovision XT system (Noldus, Leesburg, VA, USA). Time in closed arms was expressed as the percentage of time spent in the closed arms during the duration of the test and body elongation as the cumulative time the mouse stretched at least half of its body from a standing position at the center to the area of the open arms. The maze was cleaned with 10% ethanol between successive tests.

### Tissue Extraction

Mice were injected subcutaneously with ketamine (0.01 mL/g) and were decapitated after cervical dislocation. Brains were removed rapidly and frozen using liquid nitrogen. Coronal sections were cut on a Leica CM3050S cryostat using Richard-Allan Sec5e blades until the amygdala was exposed and was dissected from the caudal side. Sections were taken further on until the hippocampus area was exposed and dissected similarly. Single or multiple punches were taken to remove the area in discussion. Hippocampal punches removed the entire hippocampus, and amygdala punches removed the basolateral (BLA) area. Punches for each area from both hemispheres were pooled and stored together for each mouse in the same tube, then stored at −80°C until protein level evaluation.

### Western Blotting

#### Preparation of Cell Extracts

All protein levels were evaluated in hippocampal lysates by Western blot analysis. Lysates were made by homogenizing samples in SDS sample buffer. A volume of 15 μl of each sample was loaded into its respective well in a 26 well Criterion TGX gel (Bio-Rad, Hercules, CA, USA). Every two gels were placed in a Criterion electrophoresis cell (Bio-Rad, CA, USA) and electrophoresed under a 200 mA current for 90 min. Transfer was done using the Trans-Blot Turbo Transfer system (Bio-Rad, Hercules, CA, USA) onto 0.45 μm nitrocellulose membranes (Bio-Rad, Hercules, CA, USA). Blots were blocked in freshly prepared Tris-buffered saline solution containing 0.1% Tween 20 (TBST) with 2–5% BSA for 1 h at room temperature, with agitation. Primary antibodies and preparation: BACE Rabbit mAB #5606 (Cell Signaling, Danvers, MA, USA) diluted 1:500 in 4% BSA and 0.1% sodium azide; total-eIF2α Mouse mAB #2103 (Cell Signaling, MA, USA) diluted 1:1000 in 4% BSA and 0.1% sodium azide; phospho-eIF2α Rabbit mAB #3398 (Cell Signaling, Danvers, MA, Danvers, USA) diluted 1:1000 in 4% BSA and 0.1% sodium azide.

Each membrane was incubated overnight with its respective primary antibody, then washed in TBST three times for 5 min each. Following this series of washing steps, membranes were left for an hour incubation with a secondary antibody. Secondary antibodies were prepared in TBST solution and according to the specific primary antibody. BACE and p-eIF2α membranes were incubated in αRabbit antibody (1:10,000), whereas eIF2α membrane was incubated in αMouse antibody (1:10,000). Membranes were then developed using an XRS camera and the data analyzed by Quantity one 1-D Analysis software (Bio-Rad, Hercules, CA, USA).

#### Real Time mRNA

Total RNA (1 μg) extraction from the amygdala was performed using the RNAeasy Lipid Tissue mini kit (QIAGEN, Netherlands) according to the manufacturer’s recommendations. Reverse transcription was made using the Invitrogen Superscript III first strand synthesis (Live technologies, Carlsbad CA, USA). RNA samples were mixed with random hexamer primers and were incubated at 65°C for 5 min and briefly centrifuged. The samples then underwent a reaction using 10 μl reaction mix and then incubated again as follows: 5 min in 25°C then 50°C for 50 min. The reaction was terminated at 85°C for 5 min and was stored at −20°C. Real time RT-PCR was performed in triplicates using the ABI PRISM StepOne plus Sequence Detector (Life technologies, Grand Island, NY, USA). The analyzed genes were corticotropin releasing factor (CRF) primer #205769, CRF1 primer #7762, and NR3c1 primer #8173, all from the TaqMan Gene Expression Assays primers (Life Technologies, Grand Island, NY, USA) according to existing literature.

### Statistical Analysis

Statistical analyses were performed using GraphPad Prism6. Means of two groups were compared with a two-tailed *t* test or the Mann-Whitney-Wilcoxon U test. Comparisons of multiple groups or measurements were analyzed with one-way analysis of variance (ANOVA) or two-way ANOVA, followed by the appropriate *post hoc* tests. All groups were initially checked for normal distribution of data before proceeding with the analysis.

## Results

### High-Fat Diet Induces Changes in Anxiety Behavior Between ApoE3 and ApoE4 Mice

**Figure 1:**
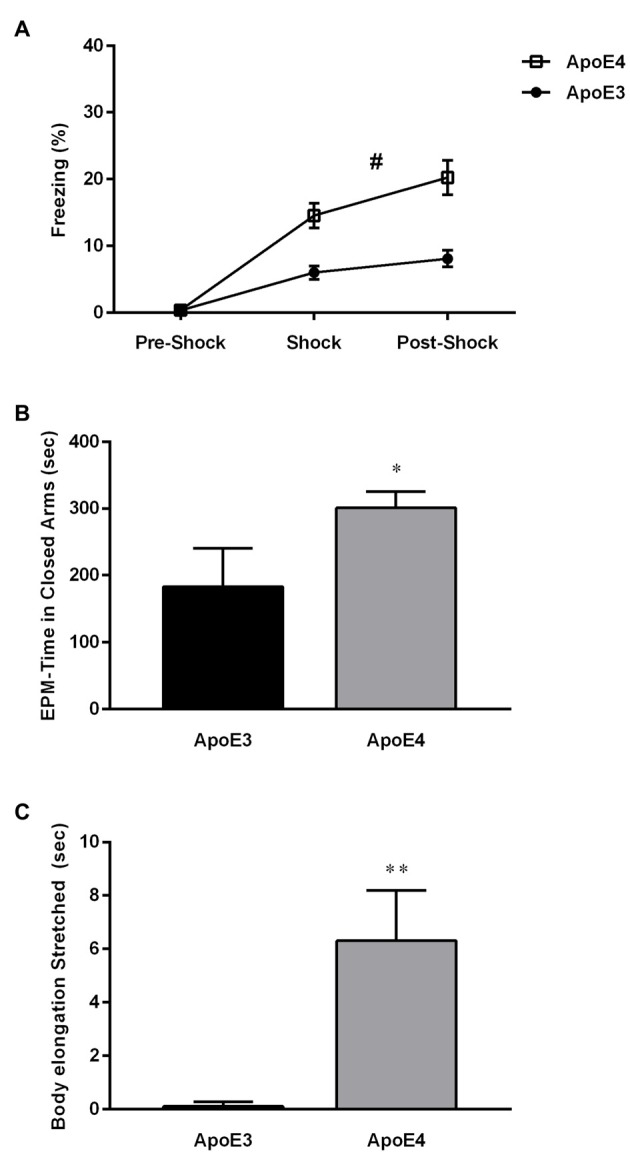
**High-fat (HF) diet induces changes in anxiety behavior between Apolipoprotein E (ApoE3) and ApoE4 mices. (A)** HF-ApoE4 and HF-ApoE3 mice were placed in an isolated chamber and after 60 s were introduced with a 2 s 0.5 mA foot shock followed by a 60 s long post-shock interval. HF-ApoE4 mice showed higher freezing time after the presentation of the foot shock, compared to HF-ApoE3 mice (Two-Way repeated measures analysis of variance (ANOVA), *F*_(1,39)_ = 17.06, ^#^*p* = 0.0002). **(B)** Four to five months old ApoE4 mice and age matched ApoE3 controls that consumed a HF diet for 20 weeks were placed in an elevated plus maze (EPM) for a period of 10 min, while their behavior was evaluated. HF-ApoE4 mice spent a longer time in the closed arms of the EPM (300.7 ± 24.37, *n* = 10) compared with ApoE3 mice (183.0 ± 57.18 *n* = 8; Mann-Whitney test, *p = 0.0341). **(C)** HF-diet mice were evaluated for cumulative duration of body elongation stretched posture. ApoE4 mice displayed a significantly longer (6.304 ± 1.880, *n* = 10) period of time in the stretched posture, compared to ApoE3 mice (0.1050 ± 0.05803, *n* = 8; *t*_(16)_ = 2.931, **p = 0.0098). Data are shown as the mean ± SEM. ^#^Relates to the repeated measuring effect of freezing between the groups *p* < 0.05.

In this study, we hypothesized that consumption of a HF diet would differentially affect ApoE4 vs. ApoE3 mice cognitively, i.e., it would further increase their metabolic burden, and as a consequence, increase differences observed in the behavioral phenotype of the hippocampal-dependent contextual memory between the two strains. For this purpose, ApoE4 (*n* = 21) and ApoE3 (*n* = 20) mice in two different batches pooled for analysis were fed on a HF diet (60% fat) for 20 weeks from weaning age. At the end of this time period the mice were tested for behavioral performance in the fear conditioning paradigm. This test was initially chosen because it had the strongest phenotype effect, however, unexpectedly, the mice showed significant differences as early as the conditioning phase of the foot shock (Figure 1A). HF-ApoE4 mice showed significantly higher freezing levels than HF-ApoE3 controls (2-way ANOVA repeated measures; *F*_(1,117)_ = 33.01, ****P < 0.0001).

These intriguing results prevented us from further testing our initial hypothesis and prompted us to propose a new one: HF diet differentially affects stress-like behavior in HF-ApoE3 vs. HF-ApoE4 mice as indicated in the higher freezing levels of HF-ApoE4 mice after presentation of the US alone. In order to test differential anxiety behavior in the two experimental groups, we used the EPM paradigm. Mice were placed on a 50 cm high plus maze placed on a table, with two open and two closed arms for a total exploration time of 10 min, during which their behavior was recorded. HF-ApoE4 (*n* = 10) mice spent significantly more time in the closed arms compared to HF-ApoE3 (*n* = 8) mice, reminiscent of stressful behavior (Hogg, 1996; Figure 1B; Mann Whitney test, *p = 0.0341). There were no differences in movement/activity between the two mouse strains (data not shown).

A more detailed analysis of behavior revealed differences in risk assessment (RA) between the two mouse strains (Blanchard et al., 1990; Griebel et al., 1995). HF-ApoE4 mice exhibited longer time spent in the stretched attended posture (SAP) compared to HF-ApoE3 mice (Figure 1C; Unpaired *t*-test, *t*_(16)_ = 2.931, **p = 0.0098), mainly at time points of aborted attempts of entry into the open arms. RA refers to a pattern of responses such as scanning, stretch attended posture, and flat back approaching (Griebel et al., 1997).

### HF-ApoE4 Mice do not Express More Stress-Induced Plasticity in the Amygdala Compared to HF-ApoE3 Controls

**Figure 2:**
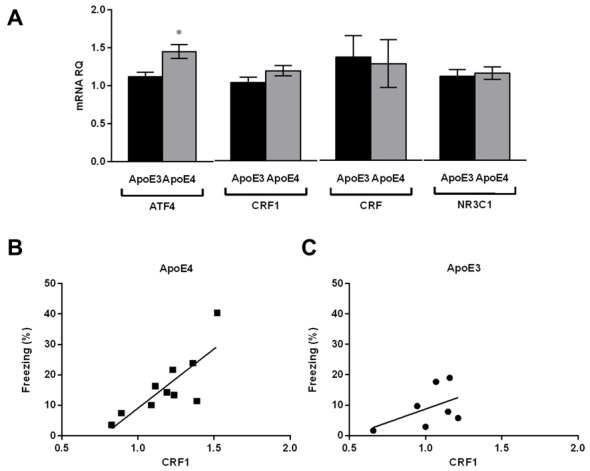
**HF-ApoE4 do not express stress-induced plasticity in the amygdala compared to HF-ApoE3 mice, but display correlation between corticotropin releasing factor 1 (CRF1) expression and freezing percent post foot-shock.** Whole amygdala was punched from the brains of mice maintained on a HF diet, and mRNA was extracted for evaluation of specific mental stress-related genes as well as the metabolic stress-related gene activating transcription factor 4 (ATF4). **(A)** There were no significant differences between HF-ApoE4 and HF-ApoE3 mice in CRF1 (*t*_(15)_ = 1.554, *p* = 0.1411), CRF (*t*_(15)_ = 0.1911, *p* = 0.8510), and NR3C1 (*t*_(15)_ = 0.3328, *p* = 0.7439). ATF4 expression was higher in HF-ApoE4 (1.449 ± 0.08855, *n* = 10) compared to HF-ApoE3 mice (1.116 ± 0.05892, *n* = 7; *t*_(15)_ = 2.831, *p = 0.0126). **(B,C)** Correlations; **(B)** HF-ApoE4 mice displayed significant correlation between amygdala CRF1 mRNA and foot shock-dependent freezing percent (*p* = 0.0052, *R*^2^ = 0.6446). **(C)** However, there was no significant correlation in HF-ApoE3 mice between amygdala CRF1 mRNA and freezing percent (*p* = 0.2656, *R*^2^ = 0.2389). Correlation was analyzed using Pearson *r* test. Data are shown as the mean ± SEM.

We next asked whether the anxiety behavior seen in young ApoE4 mice fed on the HF diet is reflected by differences in expression levels of stress-related neuropeptide mRNA in the amygdala. The CRF peptide system is critical for the behavioral response to stressful situations such as anxiety in vertebrates (Bale and Vale, 2004; Hauger et al., 2006). We thus examined the amygdala of HF-ApoE4 (*n* = 10) and HF-ApoE3 (*n* = 8) mice for CRF, CRF receptor type1 (CRF1), and glucocorticoid receptor (NR3C1) mRNA levels. We did not detect any significant changes in the above factors between the two mouse strains (Figure 2A).

We have previously shown that young (4 month) naïve ApoE4 mice express high ATF4 mRNA levels in the hippocampus compared with ApoE3 controls (Segev et al., 2015). Here, we observed that this increase is also apparent in the amygdala of the mice fed on the HF diet (Figure 2A; Unpaired *t*-test, *t*_(15)_ = 2.831, *p = 0.0126), suggesting that the increased expression levels of ATF4 in the central nervous system (CNS) of ApoE4 mice are not specific to the hippocampus. We cannot conclude from this, however, that the increased expression levels in the amygdala are due to the consumption of the HF diet, since the analysis was not performed on the amygdala of naïve mice. Interestingly, although there were no significant differences in CRF1 mRNA levels between the two mouse strains, HF-ApoE4 mice showed a strong and significant correlation between CRF1 levels and the freezing percent in response to the foot shock presentation (Figure 2B; Pearson *r* = 0.802, **p = 0.0052), whereas HF-ApoE3 mice did not show this tendency (Figure 2C; Pearson *r* = 0.488, *p* = 0.2656), suggesting that the anxiety behavior in HF-ApoE4 mice is coordinated, at least in part, by CRF receptors in the amygdala.

### ApoE4 Mice Develop Diabetes Mellitus-like Metabolic Features Following Maintenance on a High-Fat Diet

**Figure 3:**
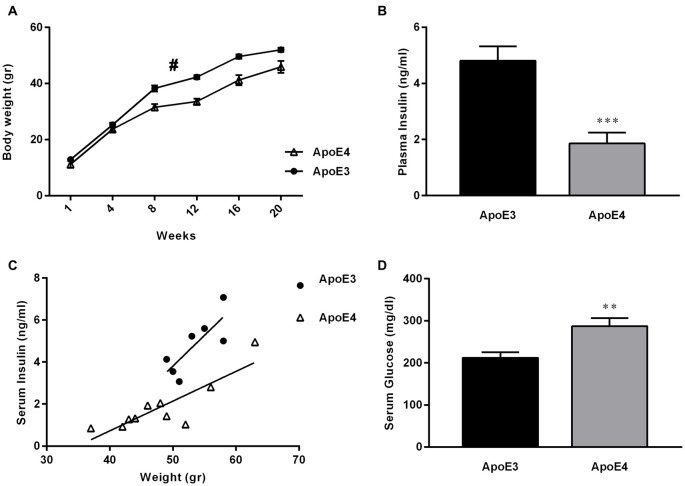
**ApoE4 mice develop diabetes mellitus (DM)-like metabolic features following maintenance on a HF diet. (A)** ApoE4 mice gained significantly less weight over a HF diet period of 20 weeks compared to ApoE3 mice (Two-way repeated measures ANOVA, strain effect; *F*_(1,16)_ = 13.54, ^#^*p* = 0.0020). **(B)** Serum-insulin was extracted and evaluated from 24 h fasted ApoE4 mice and ApoE3 controls after a 23 week HF diet consumption. HF-ApoE4 mice showed significantly lower levels of serum insulin (1.857 ± 0.3930, *n* = 10), compared with HF-ApoE3 mice (4.809 ± 0.5133, *n* = 7; *t*_(15)_ = 4.643, ***p = 0.0003). **(C)** Correlation between body weight and serum insulin was evaluated. There was a stronger correlation in HF-ApoE4 mice (*R*^2^ = 0.7317, **p = 0.0016), compared to HF-ApoE3 mice (*R*^2^ = 0.6297, *p* = 0.0332). In addition, linear regression test demonstrated significant differences between elevations or intercepts (*F*_(1,14)_ = 21.7475, ***p = 0.00036), and no differences between slopes (*F*_(1,13)_ = 2.65066, *p* = 0.1275). **(D)** Serum-glucose was extracted from non-fasting ApoE4 and ApoE3 mice after a 20 week HF diet. HF-ApoE4 mice showed significantly higher glucose levels (287.2 ± 19.25, *n* = 10) compared with HF-ApoE3 age matched controls (212.1 ± 13.25, *n* = 7; *t*_(15)_ = 2.924, **p = 0.0105). Data are shown as the mean ± SEM. ^#^Relates to the repeated measuring effect of body weight between the groups (*p* < 0.05). Correlations were analyzed using the Pearson *r* test.

Dysregulation of glucose and insulin homeostasis as well as obesity are risk factors for the development of AD (Ho et al., 2004; de la Monte and Wands, 2008; Maesako et al., 2015). Furthermore, there is growing evidence in support of the hypothesis that AD represents a form of DM that selectively affects the brain (de la Monte and Wands, 2008). Here, we confirm previous work by others (Huebbe et al., 2015), showing that ApoE4 mice fed on a HF diet gain less weight over time, compared with their HF-ApoE3 age matched controls (Figure 3A; 2-way repeated measures ANOVA, strain effect; (*F*_(1,16)_ = 13.54, **p = 0.0020).

We further demonstrate that consumption of a HF diet alone is sufficient to cause DM-like metabolic features apparent by disruption in peripheral glucose and insulin levels in the HF-ApoE4 AD mouse model (Figures 3B,C). Specifically, HF-ApoE4 (*n* = 10) mice have lower levels of peripheral plasma insulin after a 24 h starvation period (*t*_(15)_ = 4.643, ***P = 0.0003), and higher glucose levels (*t*_(15)_ = 2.924, *P = 0.0105) compared with HF-ApoE3 (*n* = 8) controls (Figure 3D). We asked whether the glucose and insulin levels in the HF-ApoE4/3 mice resemble those of C57BL/6 mice fed with the same diet (data obtained from Harlan laboratories), which would suggest whether the changes are specific to the ɛ4 allele. C57BL/6 mice fed with this diet for a period of 17 weeks show glucose levels of 146 mg/dl. Both HF-ApoE3 and HF-ApoE4 show higher levels of glucose, however, ApoE4 mice show higher levels (287 mg/dl) compared to ApoE3 mice. This hypothesis should be further evaluated by comparing the data to groups of ApoE3 and E4 fed on a normal diet. Interestingly, HF-ApoE4 mice showed a stronger correlation between body weight and peripheral serum insulin levels (Pearson *r*, **P = 0.0016) than HF-ApoE3 mice (Pearson *r*, *P = 0.033). Differences between elevations are highly significant (***p = 0.0003657).

### High-Fat Diet Promotes Changes in BACE1 Protein Expression in the Hippocampus Between ApoE3 and ApoE Mice

**Figure 4:**
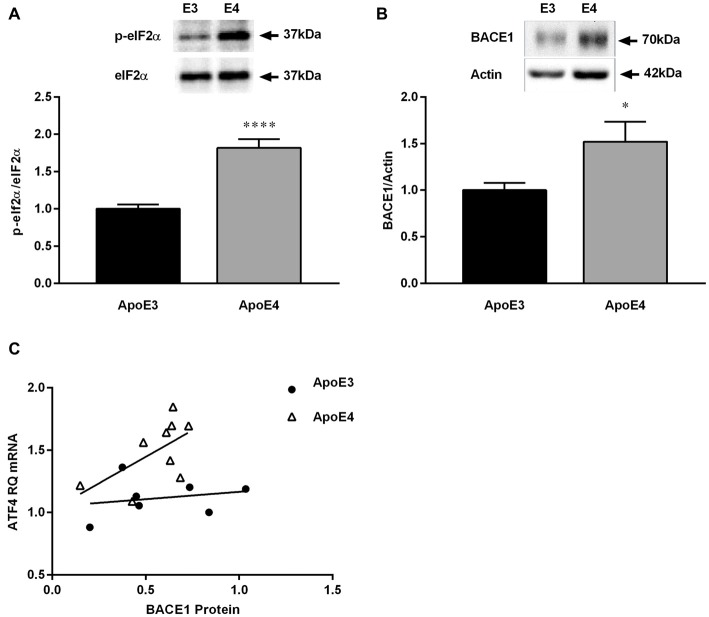
**HF diet promotes changes in β-site amyloid precursor protein-cleaving enzyme 1 (BACE1) protein expression in the hippocampus between ApoE3 and ApoE4 mice.** Whole hippocampal tissue was punched from ApoE4 and ApoE3 mice after consuming a HF diet for 23 weeks, and analyzed for protein expression. **(A)** HF-ApoE4 retained basal-high levels of eukaryotic initiation factor 2 (eIF2α)-phosphorylation in the hippocampus (1.818 ± 0.1171, *n* = 14), compared to HF-ApoE3 mice (1.000 ± 0.05911, *n* = 15, *t*_(27)_ = 6.367, ****p < 0.0001). **(B)** HF diet induced increased protein levels of BACE1 in the hippocampus of HF-ApoE4 mice (1.521 ± 0.2154, *n* = 14), compared with HF-ApoE3 mice (1.000 ± 0.07804, *n* = 13, *t*_(25)_ = 2.204, *p = 0.0369). **(C)** HF-ApoE4 mice show a stronger tendency to correlation of hippocampal BACE1 protein with amygdala ATF4 mRNA levels (*p* = 0.0920), compared with HF-ApoE3 mice (*p* = 0.6335), using the Pearson *r* test. Furthermore, differences between elevations are highly significant (*F*_(1,13)_ = 13.4541, *p* = 0.002838).

We have previously shown that phosphorylation of eIF2α is increased in 4 month old ApoE4 mice (Segev et al., 2015). As observed before in naïve mice, HF-ApoE4 mice express higher levels of eIF2α phosphorylation in the hippocampus compared with HF-ApoE3 controls (Figure 4A; *t*_(27)_ = 6.367, ****P < 0.0001). In addition, HF-ApoE4 (*n* = 10) mice express higher BACE1 protein levels in the hippocampus compared to HF-ApoE3 (*n* = 8) controls (Figure 4B; *t*_(25)_ = 2.204, *P = 0.0369).

Work by others suggests that the increase in BACE1 levels may be related to the HF consumption and the DM-like metabolic features (Patil et al., 2006; Zhang et al., 2009; Son et al., 2012), however, it has never been shown, to the best of our knowledge, in the ApoE4 AD model. There was no correlation between hippocampal BACE1 protein levels and ATF4 mRNA levels in the amygdala. This can be explained by the different measurements, i.e., mRNA vs. protein, the different tissues analyzed, i.e., amygdala vs. hippocampus, and the fact that the differences between the elevations are highly significant (Figure 4C; *F*_(1,13)_ = 13.4541, **P = 0.0028).

## Discussion

In agreement with the early emergence and slow progression of AD, environmental factors are becoming more important in our consideration of ways to delay the onset of disease symptoms. Nutrition is one such environmental factor, which is closely related to both cholesterol metabolism, i.e., ApoE genotype, and insulin/glucose impaired metabolism, i.e., DM, which are both risk factors for AD.

We have previously demonstrated that young human-targeted ApoE4 replacement mice show mild cognitive impairment (MCI) in the contextual fear conditioning paradigm, compared with ApoE3 age matched controls (Segev et al., 2013). Later publications by other groups strengthened these findings of cognitive deficiency in this mouse strain (Salomon-Zimri et al., 2014). In our previous experiments with standard diet fed mice, basal freezing levels in the conditioning phase were consistently similar between the two groups, suggesting that the unconditioned stimulus, foot shock, by itself did not cause behavioral/anxiety differences between the groups. In the present study, we asked whether a simple, noninvasive intervention such as a western-like HF diet would have an effect on the metabolism and/or cognitive behavior of an AD-relevant mouse model, the ApoE4 mice. We initially hypothesized that the cognitive impairments in young naïve ApoE4 mice (Segev et al., 2013) would be worsened by a HF diet. To this end, the AD-relevant model mice and their controls, ApoE3 mice, were given a western-like HF diet from weaning age until they reached 4 months, the age in which we previously detected behavioral abnormalities and eIF2α increased phosphorylation (Segev et al., 2013). However, unexpectedly, ApoE4 mice displayed higher anxiety levels as early as the presentation of a foot shock on the conditioning day of the fear conditioning paradigm. Due to these results the mice were not further evaluated for the memory of the fear conditioning. Instead, they were analyzed for anxiety in the EPM. The results suggest that expression of human-ApoE4 allele renders mice fed on a western-like diet more susceptible to mental stress. It should be noted that the SAP differences in behavior observed here do not seem to be necessarily related to the induction of a HF diet, since a work presented by Hartman et al. (2001) showed that 8–11 month old GFAP-ApoE4 mice fed on a normal diet do not display significant differences in the time spent in the different arms. However, these mice possess higher SAP time compared with GFAP-ApoE3 mice (Hartman et al., 2001). The results described in this study and our own suggest that ApoE4 mice have low basal levels of anxiety, which are apparent only in the SAP. However, the ApoE4 mice are prone to mental stress that can easily be triggered by environmental stimuli such as consumption of a HF diet, as indicated by higher freezing levels after presentation of a foot shock and longer time spent in the closed arms of the EPM. Conflicting evidence is apparent in regard to the level of basal anxiety in ApoE4 mice. While Johnson et al. (2014) showed differences in basal anxiety levels related to the E4 allele, Salomon-Zimri et al. (2014) showed that there were no changes in basal anxiety levels between the ApoE4 and ApoE3 mice fed on a normal diet. This could be explained either by the fact that different protocols were used in the different studies or that the basal anxiety levels are mild in normal-fed mice and therefore may not always be apparent. In any case, our results advocate that the anxiety behavior is worsened with a HF diet specifically in ApoE4 mice. For this reason the same mice (from the second batch only) were evaluated for anxiety levels using the EPM, and were not further tested for memory differences in the fear conditioning paradigm. It would be vital to evaluate the effect of genotype vs. the effect of a HF on stress behavior as well as performance on additional learning and memory paradigms. This can be done by comparison to additional groups of naïve mice fed on a normal diet. It should also be noted that a study by Siegel et al. (2012) showed that female human-ApoE4 mice express higher measures of anxiety compared to female human–apoE3 controls, and as a result, seemed to score better at the hippocampal-dependent learning of Morris water maze (MWM). On the other hand, another group detected impairments in the MWM learning paradigm in male human-ApoE4 vs. male ApoE3 mice with no apparent anxiety behavior (Salomon-Zimri et al., 2014). These conflicting results may be due to differences in protocols and gender of mice.

Our results show no changes in CRF peptide system mRNAs in the amygdala, however, these were only analyzed in non-stressed HF-ApoE mice. These mRNAs should be evaluated in mice after a stressful stimulus such as the foot shock in order to evaluate whether the stressful behavior is mirrored by changes in the CRF peptide system in the amygdala.

Work by other groups revealed that although ApoE4 mice gain less weight after consumption of a HF diet, they display dysfunctional epididymal adipose tissue, which in turn impairs the glucose tolerance in these mice (Arbones-Mainar et al., 2008). In our work, we strengthen the above by showing that ApoE4 mice indeed gain less weight over time and display abnormal peripheral glucose/insulin levels, suggesting that the ε4 allele together with an imbalanced HF diet is sufficient to induce DM-like features, which in turn may also render ApoE4 carriers more susceptible to dementia and AD (Peila et al., 2002; Yang and Song, 2013). Furthermore, the reduced body weight in the HF-ApoE4 mice may be in part due to impaired insulin-stimulated growth and survival signaling. The fact that ApoE4 mice show stronger correlation between body weight and insulin levels suggests impaired ability to balance insulin levels despite increased body weight. It should be noted that due to the fact that in the current study normal-diet fed mice were not evaluated, we cannot exclude the possibility that changes seen in the different ApoE strains were the result of changes in the ApoE3 mice and not the ApoE4 mice. For this, more groups should be included. However, existing data from previous publications, as well as metabolic data obtained from Harlan Laboratories in C57BL/6 mice, suggest that the changes are indeed specific to the E4 allele.

Genetic association studies and meta-analysis suggest an association of BACE1 and ApoE4 to impose additional risk for sporadic AD (Clarimón et al., 2003; Gold et al., 2003; Kan et al., 2005; Jo et al., 2008). In addition, studies suggest a possible role of saturated fatty acids as well as DM in the increased expression of BACE1; one group showed that conditioned medium from PA-treated astrocytes, caused BACE1 upregulation in cortical neurons (Patil et al., 2006; Zhang et al., 2009). Another group suggested that type 2 diabetes exacerbates the generation of A-beta via activation of BACE1 (Zhang et al., 2009). Importantly, we found HF treated ApoE4 mice possess increased protein levels of BACE1, the rate limiting enzyme in APP cleavage to Aβ, in the hippocampus, compared with HF-ApoE3 mice. We have never been able before to detect changes in BACE1 levels in normal diet-fed ApoE3 and ApoE4 mice (data not shown). Therefore, although the question of whether BACE1 levels are decreased in ApoE3 mice or increased in ApoE4 mice should be further studied using additional normal-diet fed groups, our previous results and the literature regarding BACE1 and obesity suggest that the changes are indeed specific to the E4 allele. Kim et al. (2011) showed that BACE1 is not altered in an allele specific manner in ApoE haploinsufficient mice, E3/E3 vs. E4/E4. This suggests that environmental factors such as nutrition in ε4 carriers affects biochemical factors closely related to the pathology of AD. In fact, in APP transgenic mice, BACE1 expression and activity were increased at sites of focal inflammation before Aβ deposition, suggesting inflammatory processes may directly promote local production of Aβ (Heneka et al., 2005).

In order to explicitly determine whether the changes in the ApoE strains were a result of the HF diet induced, further analyzing using additional groups of mice fed with a normal diet should be considered. However, we believe that essential and novel data could be concluded from our work here, which may open new horizons for studying AD progression in ε4 carriers.

In regard to the ApoE4 genotype, our results and the literature suggest that chronic metabolic burden such as inflammation may result in increased BACE1 levels, and as a result, in accumulation of Aβ peptides with time. In fact, it has been shown that ApoE4 promotes aggregation and amyloid plaque formation by displaying a weaker affinity for Aβ peptides than other ApoE variants (Liu et al., 2007). The exact mechanism by which a HF diet in combination with ApoE4 induces metabolic stress and increased BACE1 levels should be further studied. The above also strengthens the hypothesis that AD-related abnormality starts long before pathological features are apparent, and that these may be, to some extent, balanced by a healthy nutritional diet.

In the present work we demonstrate how the complex integration of genetic, metabolic and environmental factors triggers a vicious cycle having a detrimental effect on mental stress and cognitive state. In fact, environmental influences on longevity are of greatest importance. In rats, mice and non-human primates, caloric restriction prolongs life and retards the appearance of several conditions associated with aging (Bunout and Cambiazo, 1999). It would be intriguing to evaluate our new hypothesis that a balanced, healthy nutrition diet which meets the individual’s needs in regards to specific genetic and metabolic profile, may delay some of the early symptoms of AD.

## Author Contributions

YS designed the study, performed the experiments, analyzed the data, and wrote the article. AL performed the experiments and analyzed the data. MM performed the experiments and analyzed the data. KR designed the study and wrote the manuscript.

## Conflict of Interest Statement

The authors declare that the research was conducted in the absence of any commercial or financial relationships that could be construed as a potential conflict of interest. The reviewer JVS-M and handling Editor declared their shared affiliation, and the handling Editor states that the process nevertheless met the standards of a fair and objective review.
